# Use of BD BACTEC™ MGIT™ for the detection of non-tuberculous mycobacteria in sanitary water samples

**DOI:** 10.3389/fmicb.2024.1492360

**Published:** 2024-10-30

**Authors:** Vincenzo Ferraro, Francesco Bisognin, Federica Sorella, Federica Ruin, Paola Dal Monte

**Affiliations:** ^1^Department of Medical and Surgical Sciences, Alma Mater Studiorum – University of Bologna, Bologna, Italy; ^2^Microbiology Unit, IRCCS Azienda Ospedaliero-Universitaria di Bologna, Bologna, Italy

**Keywords:** non-tuberculous mycobacteria, NTM, *M. chimaera*, *M. saskatchewanense*, sanitary water, BD BACTEC MGIT Mycobacteria Growth Indicator Tubes

## Abstract

**Introduction:**

The most commonly used method for the detection of non-tuberculous mycobacteria (NTM) is culture in BD BACTEC™ MGIT™ Mycobacteria Growth Indicator Tubes incubated in an automated growth detection reader BD BACTEC™ MGIT™ 960 Instrument. The system is currently validated for the detection of mycobacteria from clinical specimens but not environmental matrices.

**Methods:**

From November 2018 to December 2023, 1,369 sanitary water samples from 92 heater–cooler units (HCUs) and 747 sanitary water samples from 489 haemodialysis instruments (dialysis) were concentrated, decontaminated, and cultured on MGIT and solid Lowenstein–Jensen media to evaluate the presence of NTM. NTM-positive cultures (*n* = 261 HCUs and *n* = 20 dialysis) were purified by Middlebrook 7H11 agar plate subcultures and identified by MALDI-TOF mass spectrometry technology.

**Results:**

The purpose of this study was to evaluate the accuracy and reproducibility of the MGIT system on sanitary water from HCU and dialysis, using the two strains most frequently isolated on these devices as sources of NTM during the Emilia- Romagna surveillance programme: *M. chimaera* (79%) and *M. saskatchewanense* (100%), respectively. To evaluate the accuracy, sanitary water was spiked with *M. chimaera* and *M. saskatchewanense* at the theoretical concentrations of 100 and 10 CFU/mL, and all resulted positive in MGIT tubes. No significant changes in time to positivity were observed when MGIT tubes were inoculated with NTM at the theoretical concentrations of 10 and 100 CFU/mL on 3 consecutive days, indicating that the detection method is reproducible.

**Discussion:**

The MGIT system is suitable for detecting the presence of NTM in sanitary water samples as it was capable of detecting up to 4 CFU/mL for both *M. chimaera* and *M. saskatchewanense*. Our results indicate that the MGIT system can be used for NTM detection not only for clinical samples but also for environmental matrices.

## Introduction

Non-tuberculous mycobacteria (NTM) are a large and heterogeneous group of acid-fast bacilli widespread in the environment; the lipid-rich hydrophobic cell wall gives mycobacteria the ability to form biofilms and survive chemical treatments used to disinfect water systems. For these reasons, NTM have emerged as the cause of waterborne opportunistic infection within healthcare facilities (Li et al., 2017). The most commonly affected patient populations are immunocompromised, post-surgical, and haemodialysis. The main routes of exposure include central venous catheters (CVC), wound exposure, and contamination during surgical procedures (Li et al., 2017; Desai and Hurtado, 2018).

Numerous studies have reported significant concentrations of mycobacteria in water systems within healthcare facilities, isolated from hospital ice machines, water-cooling systems, and haemodialysis unit water supplies (Hammer-Dedet et al., 2021; Bisognin et al., 2022).

Non-tuberculous mycobacteria belonging to *Mycobacterium avium* complex *(MAC)* such as *M. avium, M. intracellulare*, and *M. chimaera* are the most frequent cause of disseminated disease in immune-compromised patients. MAC organisms have been isolated from water samples, collected from different medical devices (Götting et al., 2016; Di Mento et al., 2019). In particular, invasive cases of *M. chimaera* infection have been associated with aerosols produced by the use of heater–cooler units (HCU) during cardiac surgery (European Centre for Disease Prevention and Control, 2016).

Recently, *M. saskatchewanense* was isolated from haemodialysis systems during an active regional surveillance programme (Bisognin et al., 2023).

The most commonly used method to detect mycobacteria in clinical samples is liquid culture, using BD BACTEC™ MGIT™ Mycobacteria Growth Indicator Tubes (MGIT tubes, Becton, Dickinson and Company, USA) supplemented with an OADC enrichment and PANTA™ solution and incubated in an automated mycobacterial detection system BD BACTEC™ MGIT™ 960 Instrument (MGIT instrument, Becton, Dickinson and Company, USA) (Tortoli et al., 1999). Before culture, the samples are decontaminated with N-acetyl-L-cysteine and sodium hydroxide (NALC-NaOH) solution.

The MGIT system has been validated by the manufacturer for the detection of mycobacteria from clinical specimens (BD, 2019). However, the ability of MGIT to grow and detect mycobacteria from environmental matrices (sanitary water) has not yet been tested.

This study aimed to evaluate the accuracy and the reproducibility of the MGIT system for NTM culture from environmental samples, using the two strains most frequently isolated from sanitary water: *M. chimaera* and *M. saskatchewanense*.

## Methods

### Study design

This study was conducted at the referral centre for the detection of mycobacteria from environmental specimens in the Emilia-Romagna Region, Microbiology Unit, IRCCS Azienda Ospedaliero-Universitaria of Bologna, Italy.

According to Emilia-Romagna surveillance programme for NTM detection, which started in November 2018, we analysed 1,369 sanitary water samples from HCUs located in cardiac surgery units and 747 from dialysis devices until December 2023.

Water samples (1 L each) were concentrated using a Microsart filtration system (Sartorius, Germany) with a cellulose nitrate membrane (0.45 mm) and resuspended in 10 mL of 0.9% saline solution (final dilution 1:100) according to ECDC guidelines (European Centre for Disease Prevention and Control, 2015). The concentrated samples were decontaminated using BBL MycoPrep solution (Becton Dickinson, USA) and resuspended in 2 mL of phosphate-buffered solution. Then, the decontaminated samples were inoculated onto a solid medium (Lowenstein- Jensen, Becton Dickinson, USA) and Middlebrook 7H9 broth (MGIT, Becton Dickinson, USA). MGIT incubation was performed on MGIT 960, a fully automated system for the rapid detection of Mycobacteria, while Lowenstein–Jensen media were incubated at 37°C, without CO2 (Clinical and Laboratory Standards Institute, 2018). Solid and liquid cultures were considered negative after 42 days of incubation without isolation of any Mycobacteria, while positive cultures were confirmed by showing the presence of NTM with Ziehl–Neelsen staining and using MALDI-TOF analysis (Bruker, Germany). MGIT culture time to positivity (TtP) was also recorded, using EpiCenter software (BD, USA).

To evaluate the performance of MGIT tubes to detect NTM in sanitary water in terms of accuracy and reproducibility, we used the two strains most frequently isolated during the active regional surveillance programme: *M. chimaera* and *M. saskatchewanense*.

Accuracy was tested by inoculating titrated NTM strains (*n* = 17 *M. chimaera* and *n* = 15 *M. saskatchewanense*) at theoretical concentrations of 100 and 10 CFU/mL into MGIT tubes and evaluating culture results.

To assess reproducibility, four *M. chimaera* strains and four *M. saskatchewanense* strains were used to spike the environmental matrix at two different theoretical concentrations (100 CFU/mL and 10 CFU/mL). These artificial samples were inoculated into MGIT tubes on 3 consecutive days.

To create artificial environmental samples, titrated NTM strains were inoculated into an environmental matrix. The environmental matrix used in this study consisted of samples of sanitary water collected from medical devices (heater–cooler units for *M. chimaera* strains and haemodialysis systems for *M. saskatchewanense* strains), which had tested negative for mycobacteria in MGIT tubes and LJ solid media.

### Preparation of the environmental matrix

Sanitary water samples of 1 litre each from different medical devices were concentrated by filtration using a 0.45-μm nitrocellulose membrane, and the collected filtrate was transferred to 50 mL tubes with the addition of 5 mL of Ringer’s solution. Decontamination was performed with N-acetyl-L-cysteine and sodium hydroxide (NALC-NaOH) solution in a 1:1 ratio, mixed by vortexing for 20–30 s and then incubated at room temperature for 15 min. The samples were neutralised by adding phosphate buffer solution BBL pH = 6.8 and then centrifuging at 2,500 g for 15 min. Pellets were resuspended in 5 mL of Ringer’s solution; 500 μL of this preparation was inoculated into MGIT tubes and 250 μL was inoculated into LJ solid media, while the remaining 4.25 mL was stored at −20°C. After 42 days of incubation, the samples that showed no growth in MGIT and LJ cultures were considered negative and pooled together to reach a final volume of approximately 100 mL of mycobacteria-negative environmental matrix from both devices (HCUs and dialysis).

### Titration of NTM strains

To prepare the inoculum, a 0.5 McFarland standard mycobacterial suspension (theoretical concentrations of 1 × 10^8^ CFU/mL) was serially diluted in sodium chloride 0.9% solution to a theoretical concentration of 10^2^ CFU/mL. Tubes containing dilutions were labelled “A” (10^8^ CFU/mL) to “G” (10^2^ CFU/mL).

To confirm the suspension titration, two 7H11 agar plates were seeded with 100 μL from tubes F and G (10^3^ and 10^2^ CFU/mL, respectively), and the number of colonies on each plate was counted after an incubation period of 2 weeks at 37°C.

To exclude bias in the estimation of concentration, plate counts in the acceptable range (25–250 CFU) were used to adjust the other one, according to the Poisson distribution (Blodgett, 2008).

### Preparation of spiked environmental samples

For the accuracy test, 15 environmental strains of *M. chimaera,* 15 environmental strains of *M. saskatchewanense*, and a standardised reference strain of *M. chimaera* DSM 44623, used in duplicate, were used to spike the NTM-negative decontaminated environmental matrix.

A volume of 900 μL of NTM-negative environmental matrix was spiked with 100 μL of mycobacterial suspension from Tube F (10^3^ CFU/mL) for a final theoretical concentration of 100 CFU/mL; the same step was repeated with Tube G (10^2^ CFU/mL) for a final theoretical concentration of 10 CFU/mL.

For the reproducibility test, two environmental strains of *M. chimaera*, the standardised reference strain *M. chimaera* DSM 44623 (in duplicate), and four environmental strains of *M. saskatchewanense* were inoculated into the NTM-negative environmental matrix.

A volume of 4.5 mL of NTM-negative environmental matrix was spiked with 500 μL of bacterial suspension from Tubes F (10^3^ CFU/mL) and G (10^2^ CFU/mL).

### Accuracy and reproducibility of MGIT system

To assess accuracy, 0.5 mL of each artificial environmental sample (*n* = 32) at 10 and 100 CFU/mL were inoculated into MGIT tubes and incubated for 42 days in the BACTEC MGIT 960.

To assess reproducibility, 0.5 mL of each artificial environmental sample (*n* = 8) at 10 and 100 CFU/mL were inoculated into MGIT tubes on 3 consecutive days and incubated for 42 days in the BACTEC MGIT 960. The samples were stored at 4°C between the inoculations.

Positive MGIT cultures were confirmed by showing the presence of NTM with Ziehl–Neelsen staining and using MALDI-TOF analysis (Bruker, Germany). MGIT culture time to positivity (TtP) was also recorded, using EpiCenter software (BD, USA).

A volume of 0.5 mL of pooled environmental matrix from different medical devices (heater–cooler units and haemodialysis systems) was inoculated into an MGIT tube and incubated in the BACTEC MGIT 960 for 42 days as a negative control.

### Statistical analysis

To evaluate the difference in terms of TtP, the Student *t*-test was performed to compare the results obtained on different settings/populations. Statistical analysis was performed using GraphPad Prism version 8.0.1 (San Diego, CA, USA). Statistical significance was set at a *p*-value of <0.05.

## Results

### NTM prevalence in sanitary water recorded during regional surveillance

**Figure 1:**
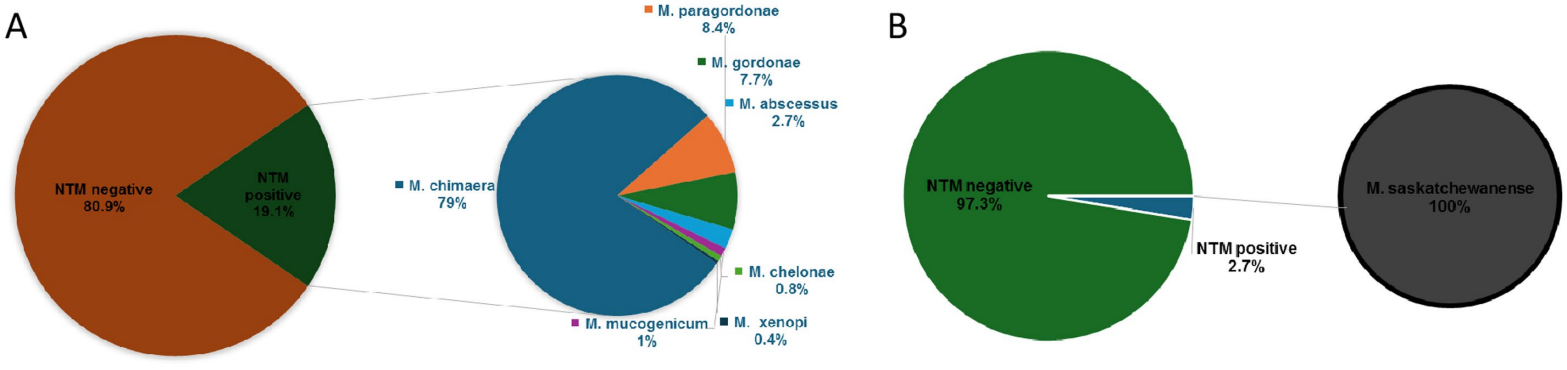
NTM prevalence during surveillance programme in HCU **(A)** and dialysis **(B)** devices.

Out of 1,369 HCU water samples collected, 261 (19.1%) were positive for NTM detection in MGIT culture: 206 (15.1%) were MGIT positive for *M. chimaera*, 22 (1.6%) for *M. paragordonae*, 20 (1.5%) for *M. gordonae*, 7 (0.5%) for *M. abscessus*, 3 (0.2%) for *M. mucogenicum, 2* (0.14%) for *M. chelonae*, and 1 (0.07%) for *M. xenopi* (Figure 1A). In contrast, out of 261 positive MGIT, solid LJ media were positive only in 71 samples. Table 1 shows NTM species detected according to liquid and solid culture results with the mean time to positivity (TtP) in MGIT. The mean TtP of MGIT culture was significantly lower (7.0 ± 1.8 days) when LJ media were positive than samples with negative LJ (18.2 ± 6.3 days) (*p* < 0.0001).

**Table 1:** Distribution of NTM-positive samples isolated from HCU, according to liquid (MGIT) and solid (LJ) cultures.

NTM-positive samples in HCU	*N* (%)	MGIT positive and LJ positive	Mean MGIT TtP ± DS (days)	MGIT positive and LJ negative	Mean MGIT TtP ± DS (days)
*M. chimaera*	206 (79%)	59	7.4 ± 1.4	147	16.4 ± 4.6
*M. paragordonae*	22 (8.4%)	0	\	22	23.8 ± 8.0
*M. gordonae*	20 (7.7%)	0	\	20	23.9 ± 8.2
*M. abscessus*	7 (2.7%)	7	3.8 ± 1.6	0	\
*M. mucogenicum*	3 (1%)	3	5.3 ± 1.1	0	\
*M. chelonae*	2 (0.8%)	1	11.7	1	16.4
*M. xenopi*	1 (0.4%)	0	\	1	28.0
Total	261	70	7.0 ± 1.8	191	18.2 ± 6.3

Out of 747 ultrapure dialysis water samples, 20 (2.7%) samples were positive for *M. saskatchewanense*, the unique NTM detected during the surveillance programme, in the study period (Figure 1B). Solid LJ media were positive in 2 (0.3%) samples, and the TtP of MGIT culture was significantly lower (9.7 ± 0.1 days) when both cultures were positive than samples with negative LJ (18.0 ± 7.8 days) (*p* = 0.01).

### Accuracy

Table 2 shows the growth and time to positivity (TtP) in liquid culture (MGIT) of artificial environmental samples at two theoretical concentrations (100 and 10 CFU/mL) of *M. chimaera* and *M. saskatchewanense*, as well as a ministerial standardised reference strain, tested twice to have a replicate.

**Table 2:** Number of colonies and time to positivity of artificial environmental samples at two concentrations.

Artificial environmental sample	Theoretical 100 CFU/mL			Theoretical 10 CFU/mL		
	Counted CFU/plate	Adjusted* CFU/plate	TtP (days)	Counted CFU/plate	Adjusted* CFU/plate	TtP (days)
*M. chimaera*-1	202	202	7.3	8	20	8.6
*M. chimaera*-2	211	211	8.4	25	21	10.3
*M. chimaera*-3	406	440	9.1	44	44	11.5
*M. chimaera*-4	181	181	9.9	9	18	14
*M. chimaera*-5	100	100	15	10	10	22.6
*M. chimaera*-6	141	140	13.9	23	14	20
*M. chimaera*-7	130	130	8.0	9	13	9.8
*M. chimaera*-8	168	168	7.1	24	16	8.5
*M. chimaera*-9	43	43	9.6	5	4	12.9
*M. chimaera*-10	296	280	7.9	28	28	9.7
*M. chimaera*-11	122	122	8.7	8	12	17.1
*M. chimaera*-12	518	460	6.1	46	46	8.1
*M. chimaera*-13	312	420	7	42	42	7.5
*M. chimaera*-14	102	102	8	9	10	8.9
*M. chimaera*-15	377	220	6	22	22	7.8
DSM 44623	94	94	8.6	10	9	11
DSM 44623	159	159	11.3	21	15	12.3
**Mean**	**210**	**204**	**8.9**	**20**	**20**	**11.8**
*M. saskatchewanense*-1	107	107	9.3	8	10	11
*M. saskatchewanense*-2	298	220	9.5	22	22	11.5
*M. saskatchewanense*-3	48	48	10.3	5	4	13.9
*M. saskatchewanense*-4	361	520	8.0	52	52	10.6
*M. saskatchewanense*-5	52	52	12.8	3	5	19.1
*M. saskatchewanense*-6	722	400	6.0	40	40	7.8
*M. saskatchewanense*-7	599	500	6.3	50	50	8
*M. saskatchewanense*-8	373	360	6.0	36	36	8.3
*M. saskatchewanense*-9	383	440	5.7	44	44	7.3
*M. saskatchewanense*-10	370	350	6.9	35	35	7.8
*M. saskatchewanense*-11	353	380	6.2	38	38	7.8
*M. saskatchewanense*-12	669	360	6.2	36	36	8.5
*M. saskatchewanense*-13	974	810	6.6	81	81	8.4
*M. saskatchewanense*-14	605	870	6.0	87	87	7.4
*M. saskatchewanense*-15	444	670	8.0	67	67	9.8
**Mean**	**460**	**406**	**7.5**	**44**	**40**	**9.7**

* Adjusted value for Poisson’s distribution; TtP, time to positivity in MGIT culture.

All artificial environmental samples spiked with NTM (*n* = 64) and inoculated in the MGIT tubes were detected as positive by the MGIT System.

Adjusted values according to Poisson distribution (range: 25–250) were used for the analysis.

For *M. chimaera* at the theoretical concentration of 100 CFU/mL, the mean number of CFU/ml detected was 204 ± 126 with a mean TtP of 8.9 ± 2.5 days, while at 10 CFU/mL, a mean of 20 ± 13 CFU/mL was detected with a mean TtP of 11.8 ± 4.4 days. The lowest concentration detected by the MGIT system was 4 CFU/mL in *M.chimaera-9* strain.

For *M. saskatchewanense* at the theoretical concentration of 100 CFU/mL, the mean number of CFU/ml detected was 406 ± 239 with a mean TtP of 7.5 ± 1.9 days, while at 10 CFU/mL, a mean of 40 ± 24 CFU/mL was detected with a mean TtP of 9.7 ± 3.0 days.

The lowest concentration detected by the MGIT system was 4 CFU/mL in *M. saskatchewanense-3* strain.

The negative control of the pooled environmental matrix inoculated into MGIT tested negative after 42 days of incubation.

### Reproducibility

Table 3 shows growth and time to positivity (TtP) in liquid culture (MGIT) of artificial environmental samples at two different theoretical concentrations (100 and 10 CFU/mL) of *M. chimaera* and *M. saskatchewanense*, as well as a ministerial standardised reference strain, tested twice to have a replicate.

**Table 3:** Number of colonies and time to positivity of artificial environmental samples inoculated into MGIT tubes on 3 consecutive days.

Artificial environmental sample	Theoretical 100 CFU/mL		TtP (days)			Theoretical 10 CFU/mL		TtP (days)		
	Counted CFU/plate	Adjusted* CFU/plate	1st day	2nd day	3rd day	Counted CFU/plate	Adjusted* CFU/plate	1st day	2nd day	3rd day
*M. chimaera*-16	190	350	7.2	7.9	8.6	35	35	8.8	9.1	9.6
*M. chimaera*-17	119	119	7.9	7.3	7.6	15	12	8.1	8.6	9.6
DSM 44623 a	295	510	8.6	8.6	8.4	51	51	10.6	9.5	9.3
DSM 44623 b	426	400	8.8	9.8	10.3	40	40	11	10.4	10.7
**Mean**	**268**	**345**	**8.1**	**8.4**	**8.7**	**35**	**34**	**9.6**	**9.4**	**9.8**
*M. saskatchewanense*-16	585	560	6.7	6.4	6.4	56	56	8.5	8.8	8.7
*M. saskatchewanense*-17	416	530	6.5	7	7.3	53	53	9.1	8.9	10.3
*M. saskatchewanense*-18	720	570	7.6	7.4	9.3	57	57	9.4	9.7	11.9
*M. saskatchewanense*-19	987	1,450	6.5	6.9	7.0	145	145	8.3	8.5	9.6
**Mean**	**677**	**777**	**6.8**	**6.9**	**7.5**	**78**	**78**	**8.8**	**9.0**	**10.0**

* Adjusted value for Poisson’s distribution.

Growth was detected in all spiked artificial samples (*n* = 48) incubated in the MGIT system for 3 consecutive days.

Adjusted values according to Poisson distribution (range: 25–250) were used for the analysis.

The mean TtP of MGIT for artificial samples with a mean of 345 ± 165 CFU/mL of *M. chimaera* on 3 consecutive days was 8.1 ± 0.7, 8.4 ± 1, and 8.7 ± 1.1 days. When a mean of 35 ± 16 CFU/mL of *M. chimaera* was inoculated on 3 consecutive days, the mean TtP was 9.6 ± 1.3, 9.4 ± 0.75, and 9.8 ± 0.58 days.

The mean TtP of MGIT for artificial samples with 777 ± 449 CFU/mL of *M. saskatchewanense* on 3 different days was 6.8 ± 0.5, 6.9 ± 0.4, and 7.5 ± 1.2 days, while at 78 ± 45 CFU/mL, the average TtP was 8.8 ± 0.5, 9.0 ± 0.5, and 10.0 ± 0.3 days.

**Figure 2:**
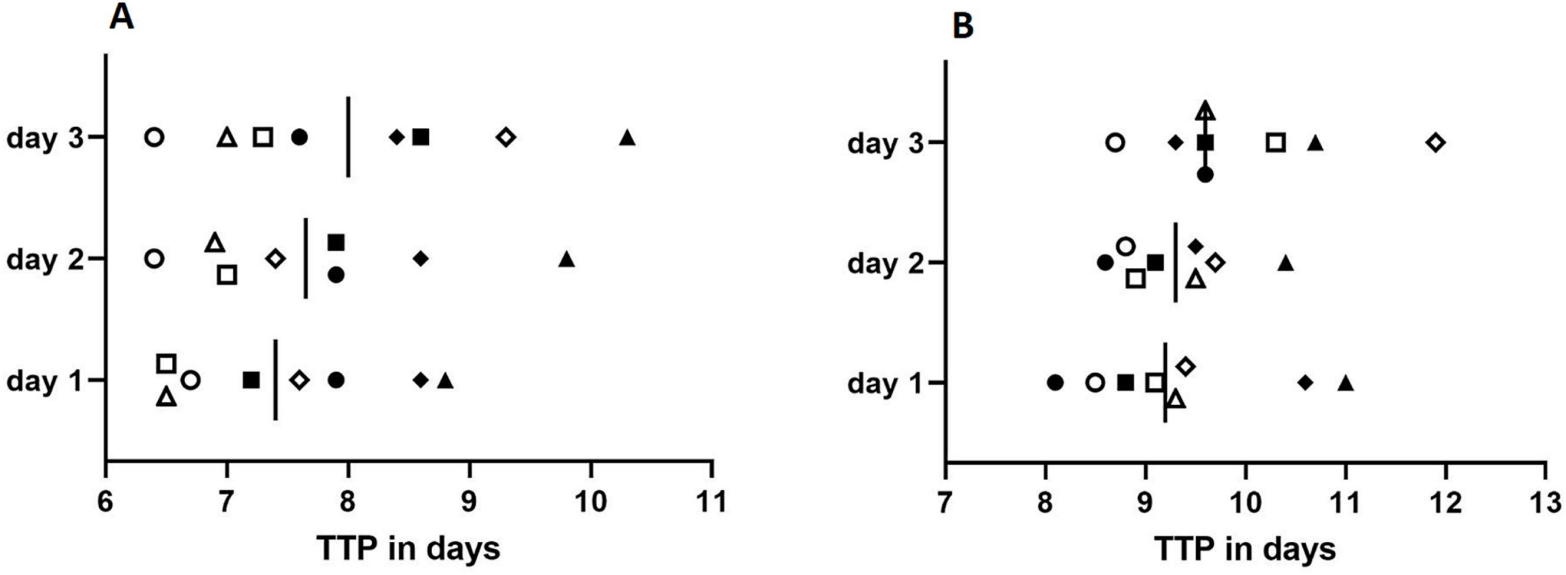
Reproducibility assay performed at the concentration of 100 CFU/mL **(A)** and 10 CFU/mL **(B)**. Full shapes depict artificial environmental samples spiked with *M. chimaera* (square*: M. chimaera*-16; circle: *M. chimaera*-17; and rhombus and triangle: *DSM 44623* in duplicate), while empty shapes depict artificial environmental samples spiked with *M. saskatchewanense* (square*: M. saskatchewanense-17*; circle: *M. saskatchewanense-16*; rhombus: *M. saskatchewanense-18,* and triangle: *M. saskatchewanense-19*). Vertical bars represent the mean of time to positivity (TtP) in liquid culture.

Figure 2 shows TtP in liquid culture of the eight samples of *M. chimaera* and *M. saskatchewanense* inoculated on 3 consecutive days at the theoretical concentrations of 100 CFU/mL (Figure 2A) and 10 CFU/mL (Figure 2B).

The comparison of TtP (days) obtained from all MGIT inoculated on 3 consecutive days showed no statistical significance with the Student *t*-test for all samples (*p* ˃ 0.05).

## Discussion

Over the last few decades, increasing evidence has showed that non-tuberculous mycobacteria (NTM) are present in hospital water systems, making them a reservoir of risk for healthcare-associated infection (Bisognin et al., 2022; Bisognin et al., 2023; Cervia et al., 2008; Sartori et al., 2013; van Ingen et al., 2017). They can cause a variety of diseases, especially among immunocompromised hosts (Li et al., 2017; Desai and Hurtado, 2018).

For this reason, the spread of NTM represents a public health problem that requires specific surveillance programmes to limit diffusion and healthcare facility-associated infections (Castro-Rodriguez et al., 2024).

According to these recommendations, from November 2018, the Emilia-Romagna Region started an active surveillance programme for the detection of NTM from environmental specimens and identified our laboratory as a regional referral centre.

We analysed a large number of sanitary water samples during the study period: 1369 from heater–cooler units (HCUs) in cardio-surgery rooms and 747 from haemodialysis monitors. The most frequent NTM detected during surveillance on HCU was *M. chimaera* (79% of NTM-positive samples). *M. paragordonae* and *M. gordonae*, mycobacteria with a low pathogenic significance, represented 8.4 and 7.7% of NTM-positive samples, respectively. The isolation of *M. abscessus*, *M. mucogenicum*, *M. chelonae,* and *M. xenopi* was a rare event.

In contrast, the unique NTM detected during monitoring of haemodialysis instruments was *M. saskatchewanense*, a recently described slowly growing scotochromogenic species, misidentified as *M. intracellulare* (Bisognin et al., 2023; Cannas et al., 2024).

MGIT culture associated with N-acetyl-L-cysteine and sodium hydroxide (NALC-NaOH) decontamination is currently the most commonly used method to detect mycobacteria from clinical specimens (Tortoli et al., 1999). The majority of the NTM species can grow in this liquid medium (Tortoli et al., 1999); however, international guidelines suggest to use also a solid medium to increase culture sensibility (European Centre for Disease Prevention and Control, 2015; Tenover et al., 1993). During our surveillance programme, we noticed that among positive MGIT cultures for NTM, only a few water samples (73/281, 30%) were also positive in LJ culture. These samples showed a lower time to positivity (TtP) than samples with negative LJ, indicating that only the highest mycobacterial load was detected in this solid medium. These results confirm that MGIT is more sensitive than LJ to detect mycobacteria (Tortoli et al., 1999).

To date, however, the performance of MGIT on environmental samples has not been validated; the main concern regards the presence of residues of disinfectants used to sanitise the devices, which could interfere with the detection of mycobacteria.

This is the first study to assess the accuracy and reproducibility of the MGIT culture system to detect NTM in sanitary water from HCU and haemodialysis medical devices.

For this purpose, we chose the same theoretical concentration adopted by the manufacturers (BD) for the validation of clinical samples using inoculum levels ranging from 10^1^ to 10^2^ CFU/mL (BD, 2019). As a source of NTM, we used a high number of *M. chimaera* and *M. saskatchewanense* strains, being the most frequently isolated NTM in sanitary water from HCU and haemodialysis, respectively.

This study showed that the MGIT system is highly sensitive in detecting NTM from sanitary water as all tubes inoculated with artificial environmental samples exhibited growth and concentrations as low as 4 CFU/mL were detected. Furthermore, the MGIT system was highly reproducible; no statistical difference in time to positivity was observed between artificial environmental samples inoculated on 3 consecutive days.

In conclusion, MGIT could be used as a liquid growth medium for the detection of NTM in environmental samples, to monitor contamination in medical equipment such as heater–cooler units, endoscope reprocessing devices, or haemodialysis systems, to prevent patient infection. This study could be useful to draft guidelines for the detection of NTM on environmental matrices.

## Data Availability

The raw data supporting the conclusions of this article will be made available by the authors, without undue reservation.
